# Untargeted sequencing of circulating microRNAs in a healthy and diseased older population

**DOI:** 10.1038/s41598-022-06956-4

**Published:** 2022-02-22

**Authors:** Lukas Streese, Philippe Demougin, Paula Iborra, Alexander Kanitz, Arne Deiseroth, Julia M. Kröpfl, Arno Schmidt-Trucksäss, Mihaela Zavolan, Henner Hanssen

**Affiliations:** 1 Department of Sport, Exercise and Health, Medical Faculty, University of Basel, Birsstrasse 320 B, 4052 Basel, Switzerland; 2 Transfaculty Research Platform Molecular and Cognitive Neurosciences, Life Sciences Training Facility, Biozentrum, University of Basel, Basel, Switzerland; 3 Computational and Systems Biology, Biozentrum, University of Basel, Basel, Switzerland

**Keywords:** Biomarkers, Physiology, Cardiovascular biology, Metabolism

## Abstract

We performed untargeted profiling of circulating microRNAs (miRNAs) in a well characterized cohort of older adults to verify associations of health and disease-related biomarkers with systemic miRNA expression. Differential expression analysis revealed 30 miRNAs that significantly differed between healthy active, healthy sedentary and sedentary cardiovascular risk patients. Increased expression of miRNAs miR-193b-5p, miR-122-5p, miR-885-3p, miR-193a-5p, miR-34a-5p, miR-505-3p, miR-194-5p, miR-27b-3p, miR-885-5p, miR-23b-5b, miR-365a-3p, miR-365b-3p, miR-22-5p was associated with a higher metabolic risk profile, unfavourable macro- and microvascular health, lower physical activity (PA) as well as cardiorespiratory fitness (CRF) levels. Increased expression of miR-342-3p, miR-1-3p, miR-92b-5p, miR-454-3p, miR-190a-5p and miR-375-3p was associated with a lower metabolic risk profile, favourable macro- and microvascular health as well as higher PA and CRF. Of note, the first two principal components explained as much as 20% and 11% of the data variance. miRNAs and their potential target genes appear to mediate disease- and health-related physiological and pathophysiological adaptations that need to be validated and supported by further downstream analysis in future studies.

Clinical Trial Registration: ClinicalTrials.gov: NCT02796976 (https://clinicaltrials.gov/ct2/show/NCT02796976).

## Introduction

Circulating microRNAs (miRNAs) are small non-coding nucleotide sequences that regulate cell function^1^. They are post-transcriptional regulators of several processes like inflammation, oxidative stress and lipid metabolism, which contribute to the progression of atherosclerosis^2^. Previous studies have highlighted the potential of circulating miRNAs as new biomarkers of cardiovascular (CV) risk and novel treatment targets in personalized medicine^3,4^. A large cohort study of 1112 coronary artery disease patients identified three circulating miRNAs (miR-132, miR-140-3p and miR-210) that significantly improved the prediction of cardiovascular mortality^5^. De Rosa et al. demonstrated that specific cardiac miRNAs (miR-499 and miR-133a) were released from the heart into the coronary circulation in coronary artery disease patients with myocardial injury^6^. Cardiac specific miRNAs can also differentiate between non-ischaemic and ischaemic heart failure^7^, which highlights the diagnostic potential of circulating miRNAs as sensitive biomarkers for manifest CV disease^8^. The ESC Working Group of Atherosclerosis and Vascular Biology highlighted circulating miRNAs as promising biomarkers for several CV diseases but discussed the low evidence level for circulating miRNAs to detect early stages of atherosclerosis^9^. Fichtlscherer et al. summarized potential sensitive circulating miRNAs in patients with vascular disease^10^. Especially the endothelium-enriched miR-126, known for its vasoprotective function^11–13^, was found to be downregulated in patients with stable coronary artery disease^14^ and was also associated with type 2 diabetes^15^.

Circulating miRNAs involved in various non-communicable diseases such as CV disease, lung disease or cancer have been shown to be affected by exercise^16–18^. We have previously shown that DNA methylation of the p66^Shc^ gene promoter may represent a putative mechanistic link by which physical activity (PA) and cardiorespiratory fitness (CRF) protect against age-related oxidative stress, an important mechanism for healthy vascular ageing^19^. The identification of miRNAs associated with individual CV risk profiles and vascular function as well as physical activity and fitness may provide new epigenetic insights into exercise-related vasoprotection and risk reduction^20^. In a translational approach, we aimed to identify and unravel associations of upstream gene regulation with downstream manifestation of end organ phenotype by sequencing of circulating miRNAs in combination with sensitive cardiovascular phenotyping. To achieve this, we performed untargeted profiling of circulating miRNAs in the well characterized EXAMIN AGE cohort involving physically active and inactive, healthy and diseased older adults.

## Methods

Next generation sequencing of stored blood serum from the “Exercise, Arterial Crosstalk-Modulation, and Inflammation in an Aging Population (EXAMIN AGE)” study^21^, were performed to investigate the association between circulating miRNAs and the cardiometabolic and vascular parameters in healthy active (HA) and healthy sedentary (HS) individuals as well as sedentary patients at increased CV risk (SR). The study was performed at the Department of Sport, Exercise and Health in Basel, Switzerland. miRNA isolation, RNA quality control, library preparation and sequencing were performed by the Life Sciences Training Facility of the Biozentrum at the University of Basel, Switzerland. The computational analysis was performed by the Computational and Systems Biology group of the Biozentrum at the University of Basel, Switzerland. The study was planned and conducted in accordance with the protocol and principles stated in the Declaration of Helsinki^22^. The Ethics Committee of Northwest and Central Switzerland (EKNZ 2015-351) approved this study. All individuals signed a written informed consent. The inclusion and exclusion criteria, blood sampling, miRNA isolation, RNA quality control, library preparation, clustering and sequencing as well as the computational analysis methods are explained in detail below. A detailed study protocol with extensive method descriptions of clinical and vascular assessments has previously been published^21^.

### Inclusion and exclusion criteria

Men and women aged 50–80 years were recruited between January 2016 to December 2017 through newspaper advertisements and local sports clubs. Inclusion criteria for HA were a physically active lifestyle (> 9 metabolic equivalents [METs]/week). HS and SR were characterized by an inactive lifestyle (≤ 3 METs/week). HA and HS were assessed as healthy (without any of the CV risk factor described in Supplementary Table S1). SR patients were selected to have ≥ 2 CV risk factors. Exclusion criteria for HA and HS individuals were a history of CV disease, pulmonary or chronic inflammatory disease, any CV risk factor described in Supplementary Table S1, or any chronic eye disease. Exclusion criteria for SR patients were decompensated cardiopulmonary disease or chronic inflammatory disease, and chronic eye disease.

PA behaviour was screened based on PA history, self-reported Freiburg Questionnaire of Physical Activity (FQPA), accelerometer (ACC) data and maximal oxygen uptake (VO_2_peak). Participants wore the ACC for six consecutive days. The five most active days were analysed. Two sports scientists independently judged the level of PA and decided to allocate individuals either into the active or sedentary group or to exclude the subject.

### Blood sampling

Blood samples were drawn by trained medical staff and venepuncture of the cubital fossa of the right or left arm. Serum was centrifuged at 2000×*g* for ten minutes, isolated and stored at − 80 °C. Blood samples were collected in a fasting state. Participants were not allowed to exercise 24 h and smoke or drink high energy drinks or coffee 6 h before blood sampling.

### miRNA isolation

Blood serum was centrifuged at 3000×*g* for 30 min at 4 °C after defrosting. 250 μl serum were processed for small RNA isolation using miRNeasy Serum/Plasma Advanced Kit (Qiagen, Cat# 217204). Elution was performed in 20 μl.

### RNA quality control

1.5 μl of eluate was used for assessing the quality of isolation, the absence of inhibitor for enzymatic reaction, and a group of endogenous controls by real-time polymerase chain reaction (PCR). More precisely, the kits QIAseq miRNA Library QC qPCR Assay Kit (Qiagen, Cat# 331551), miRCURY LNA miRNA PCR Assay (Qiagen, Cat# 339306-YP00203907) and miRCURY LNA SYBR Green PCR Kit (Qiagen, Cat# 339347) were used.

### Library preparation

5 μl of eluate was used to perform library preparation by series of 48 samples, using the QIAseq miRNA Library Kit (96) (Qiagen, Cat# 331505) and the QIAseq miRNA 48 Index IL, 96 (Qiagen, Cat# 331595). 22 cycles of PCR were performed. Libraries were quality-checked on the Fragment Analyzer (Advanced Analytical, Ames, IA, USA) using the High Sensitivity NGS Fragment Analysis Kit (Cat# DNF-474, Advanced Analytical, Ames, IA, USA) revealing excellent quality of libraries.

### Small RNA sequencing

Samples were sequenced as single-read, 76 bases using the NextSeq 500 High Output Kit 75-cycles (Illumina, Cat# FC-404-1005). Primary data analyses were performed with the Illumina RTA version 2.4.11 and Basecalling Version bcl2fastq-2.20.0.422.

### miRNA sequence analysis

miRNA reads were processed with a custom workflow developed by the Zavolan lab and described previously^23^. In brief, after removing adaptor sequences, reads with less than 15 bases in length were discarded, reads with the same sequence were collapsed and resulting unique reads were mapped to the human genome GRCh38 assembly version and corresponding transcriptome release (ENSEMBL release 96) using Segemehl^24^ and Oligomap^23^ tools. Alignments were intersected with gene (ENSEMBL release 96) and miRNA precursor (miRBase v22.1) annotations. Reads aligning to loci of miRNA precursors were retained and counted either towards the corresponding miRNAs' 5p (first half of the precursor) or 3p (second half of the precursor) mature miRNAs. Reads that mapped to N miRNA loci were counted as 1/N towards each of the loci. A summary table containing miRNA read counts for all samples was created and used as input for further analysis using the R software.

### Statistical analysis

The statistical software R (version 4.0.5) was used for all statistical analyses. Descriptive statistics were used to characterise the miRNA samples. To identify differential miRNA expression between groups the edgeR^25^ package (version 3.32.1) was used. A filtering strategy was implemented to retain only those miRNAs that were found expressed in a minimum number of samples. To only consider miRNAs that are expressed at reasonably high levels across a considerable number of samples, we have applied edgeR’s filterByExpr () function with default parameters. This led to the selection of 299 miRNAs, whose normalized read count (read count/median sample size * 106) was at least 10 in at least 70% of the samples of the smallest group (which was HS). To obtain normalized miRNA counts, the calcNormFactors () function was used applying the trimmed mean of M-values (TMM) method. The log2-fold-change, logFC, the average log2-counts-per-million, (logCPM), and the two-sided P-values were then computed using the exactTest() function for each group comparison. The miRNAs with significant differences in expression between groups were extracted and the false discovery rates (FDR) were calculated based on the *P* values using the Benjamini–Hochberg method. An FDR threshold of 0.05 was used to identify miRNAs whose expression changed significantly. Spearman’s rank correlation coefficients were calculated to identify miRNAs whose expression levels were correlated to specific physiological parameters. Molecular and physiological parameters were summarized for all individuals, and principal component analysis was used to identify the main directions of variation in the data and the parameters most responsible for this variation. The principal component analysis was carried out with the prcomp() function of R, applied to log-transformed miRNA expression values and physiological parameters, centered and scaled across both parameters and patients. The contributions of various features to the principal components were obtained with the function fviz_pca_var() from the factoextra package version 1.0.7. We used target predictions of the MIRZA-G algorithm which we developed^26^, and we cross-checked the filtered target genes with a commonly used database (TargetScan version 7.2). We used pairwise tests for miRNA expression changes between group conditions. Analysis of variance was applied to investigate overall group differences of sample characteristics. The sample size was based on the estimated group differences in arterial stiffness as the primary outcome as described in the study protocol^21^. Samples from all participants taking part in the original EXAMIN AGE study were analysed for the current study.

## Results

One hundred fifty-eight participants were included in this study (Supplement Fig. S1), allocated to HA (n = 38), HS (n = 36) and SR (n = 84) groups. Sample characteristics are described in Table 1. The CV profiles of HA and SR were comparable whereas SR showed a higher CV risk profile. HA showed higher PA and CRF levels compared to both sedentary groups (Table 1). HA were characterized by a lower pulse wave velocity and a higher arteriolar-to-venular diameter ratio (AVR), both associated with better vascular health, compared to HS with a further decline in SR (Table 1).

**Table 1:** Sample characteristics.

	HA (n = 38) mean (SD)	HS (n = 36) mean (SD)	SR (n = 84) mean (SD)	*P*
**Patients’ characteristics**				
Sex (f/m)	17/21	26/10	42/42	0.036
Age (years)	60 (7)	60 (7)	59 (6)	0.570
Height (cm)	171.1 (7.7)	167.5 (8.8)	168.9 (8.0)	0.160
Weight (kg)	64.5 (6.5)	70.2 (9.9)	94.7 (14.0)	< 0.001
BMI (kg/m^2^)	22.1 (1.7)	24.8 (2.4)	33.2 (4.1)	< 0.001
WC (cm)	82.1 (6.6)	89.4 (8.9)	111.4 (11.5)	< 0.001
HC (cm)	86.5 (4.9)	95.2 (6.9)	112.0 (10.0)	< 0.001
Fat mass (kg)	13.0 (3.8)	22.8 (5.9)	37.9 (9.7)	< 0.001
Muscle mass (kg)	28.6 (4.4)	25.9 (4.8)	31.6 (6.9)	< 0.001
24 h systolic BP (mmHg)	120 (6)	121 (7)	130 (11)	< 0.001
24 h diastolic BP (mmHg)	76 (5)	76 (6)	81 (8)	< 0.001
Fasting glucose (mmol/l)	4.7 (0.4)	4.7 (0.5)	5.8 (1.8)	0.014
Triglyceride (mmol/l)	0.9 (0.3)	1.1 (0.3)	1.8 (1.1)	0.002
HDL (mmol/l)	2.0 (0.4)	1.7 (0.4)	1.3 (0.3)	< 0.001
LDL (mmol/l)	3.0 (0.7)	3.2 (0.8)	3.2 (0.8)	0.535
IL10 (pg/ml)	0.09 (0.24)	0.14 (0.37)	0.10 (0.18)	0.717
IL6 (pg/ml)	0.85 (2.90)	0.96 (1.31)	2.47 (2.02)	< 0.001
TNF alpha (pg/ml)	0.50 (0.80)	1.72 (1.73)	1.25 (1.60)	0.002
Hs-CRP (mg/l)	0.94 (0.97)	2.01 (2.92)	3.58 (4.10)	< 0.001
IOP (mmHg)	16 (3)	16 (3)	17 (3)	0.291
**Activity and fitness**				
ACC_active counts (n)	11,193 (4171)	11,363 (4012)	9987 (4069)	0.142
ACC_steps per day (n)	13,267 (4869)	10,105 (3828)	8711 (3588)	< 0.001
ACC_walking per day (min)	148 (52)	121 (44)	105 (43)	< 0.001
ACC_distance per day (m)	9310 (3385)	6424 (2696)	5567 (2398)	< 0.001
ACC_sportive per day (min)	7.9 (10.6)	0.3 (1.3)	0.1 (0.5)	< 0.001
VO_2_peak (ml/min/kg)	42.5 (8.3)	29.9 (4.3)	26.0 (4.3)	< 0.001
**Vascular health**				
PWV (m/s)	7.0 (1.1)	7.5 (1.6)	8.2 (1.4)	< 0.001
24 h PWV (m/s)	8.4 (1.2)	8.4 (1.1)	8.5 (1.0)	0.797
CRAE (µm)	179 (14)	172 (11)	171 (14)	0.026
CRVE (µm)	204 (17)	209 (11)	218 (16)	< 0.001
AVR	0.88 (0.05)	0.83 (0.04)	0.79 (0.05)	< 0.001

*HA* healthy active, *HS* healthy sedentary, *SR* sedentary at risk, *SD* standard deviation, *p* ANOVA level of significance for overall group differences, *BMI* body mass index, *WC* waist circumference, *HC* hip circumference, *BP* blood pressure, *HDL* high-density lipoprotein, *LDL* low-density lipoprotein, *IL* interleukin, *TNF* tumor necrosis factor, *Hs-CRP* high sensitive C-reactive protein, *IOP* intraocular pressure, *FQPA* Freiburg questionnaire of physical activity, *METs* metabolic equivalents, *ACC* accelerometer, *VO2peak* peak oxygen uptake, *PWV* pulse wave velocity, *CRAE* central retinal arteriolar equivalent, *CRVE* central retinal venular equivalent, *AVR* arteriolar-to-venular diameter ratio.

### Expression profiles of annotated miRNAs in blood samples

**Figure 1:**
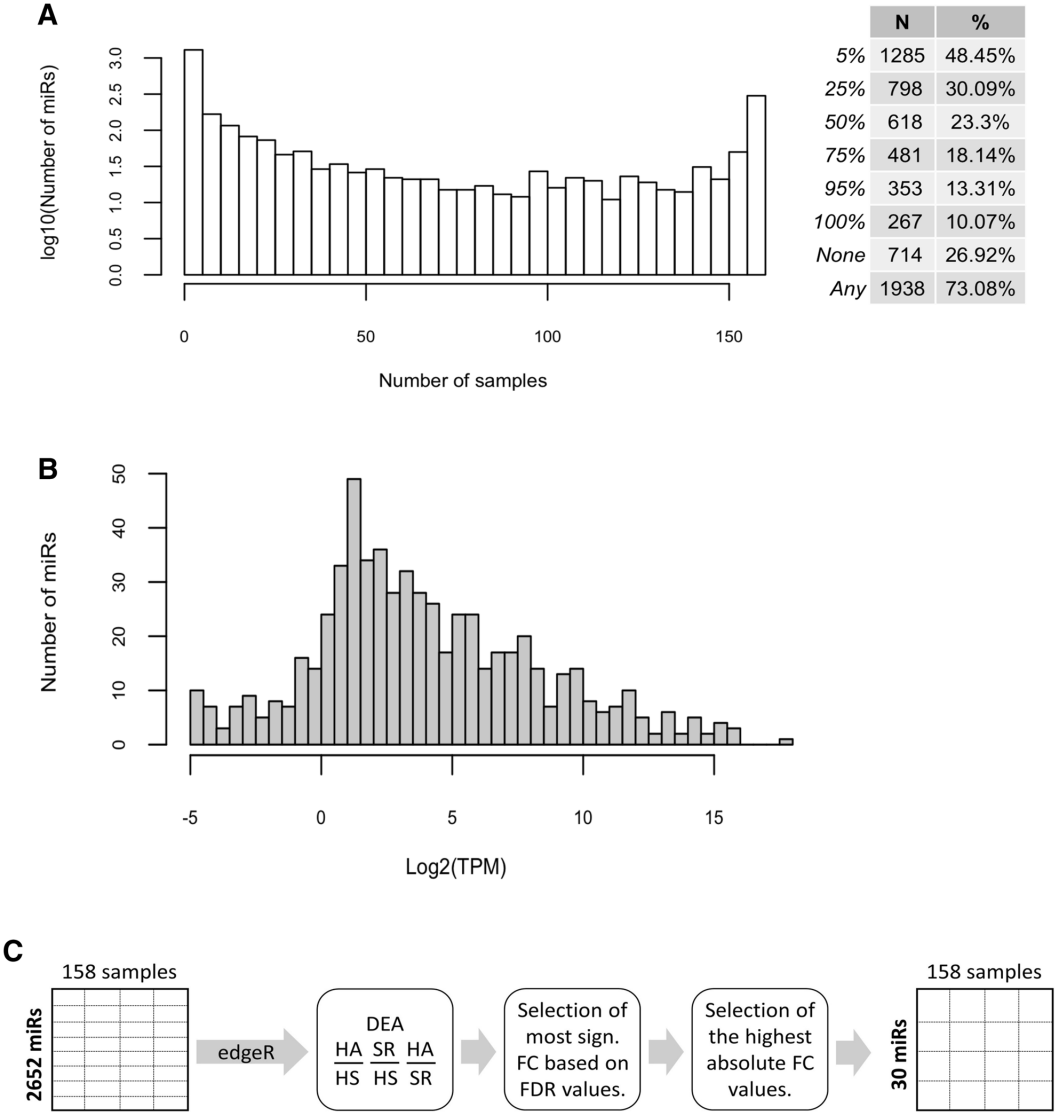
Summary of analysis of EXAMIN AGE data. (**A**). Distribution of the number of individual miRNAs detected in specific numbers of samples and number and percentage of mature miRs identified in the indicated proportions of samples. (**B**). Numbers of miRNAs with specific values of median expression across samples (miRNAs with zero median expression are not shown). (**C**). Outline of the comparison of miRNA expression between groups of patients. *DEA* differential expression analysis, *HA* healthy active, *HS* healthy sedentary, *SR* sedentary at increased CV risk, *FC* fold change, *FDR* false discovery rate.

**Figure 2:**
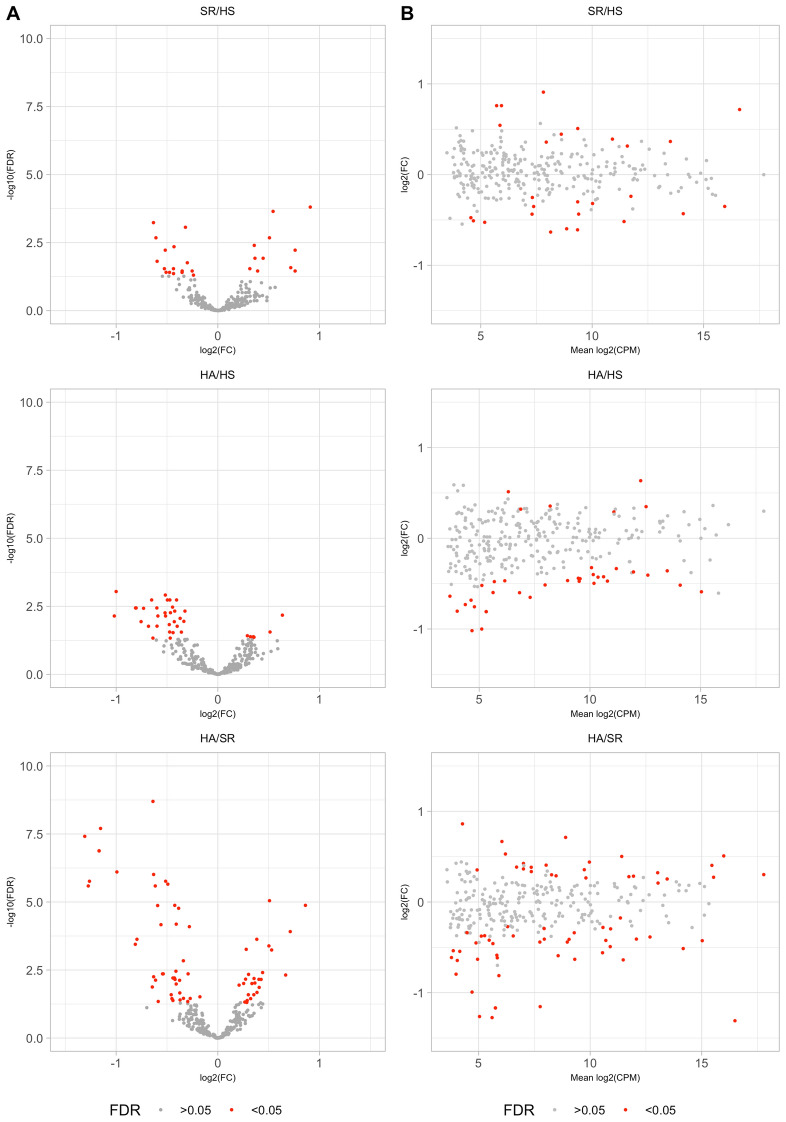
miRNA expression changes between conditions. (**A**) ‘Volcano plots’ showing the fold-change versus statistical significance measure for pairs of sample groups. (**B**) Mean versus fold-change expression of individual miRNAs in pairs of sample groups. *HA* healthy active, *HS* healthy sedentary, *SR* sedentary at increased CV risk, *FDR* false discovery rates, *CPM* count-per-million.

Investigating the distribution of the number of distinct miRNAs detected in individual samples we found that most (27%) of the 1938 annotated miRNAs were either not detected in any sample, or were detected in all samples (10%, Fig. 1A). While the median expression of a miRNA across samples was generally low (2–4 counts per million miRNA reads in a sample, there were also miRNAs that were present in 10^3^–10^4^ copies per million miRNA in a sample (Fig. 1B). Finally, we carried out a differential expression analysis (Fig. 1C) to identify a set of 30 miRNAs whose expression differed significantly between HA, HS and SR (Fig. 2). As shown in Fig. 2, while the HS and SR groups exhibited relatively small differences, a group of miRNAs showed reduced expression in HA relative to both HS and SR (Fig. 2A). These miRNAs covered the entire expression range (Fig. 2B). Interestingly, while differences in miRNA expression were already detectable in the HA versus HS comparison, they were much more pronounced in the HA versus SR comparison (Fig. 2A).

### Correlation of miRNA expression with biomarkers of cardiovascular health and disease

**Figure 3:**
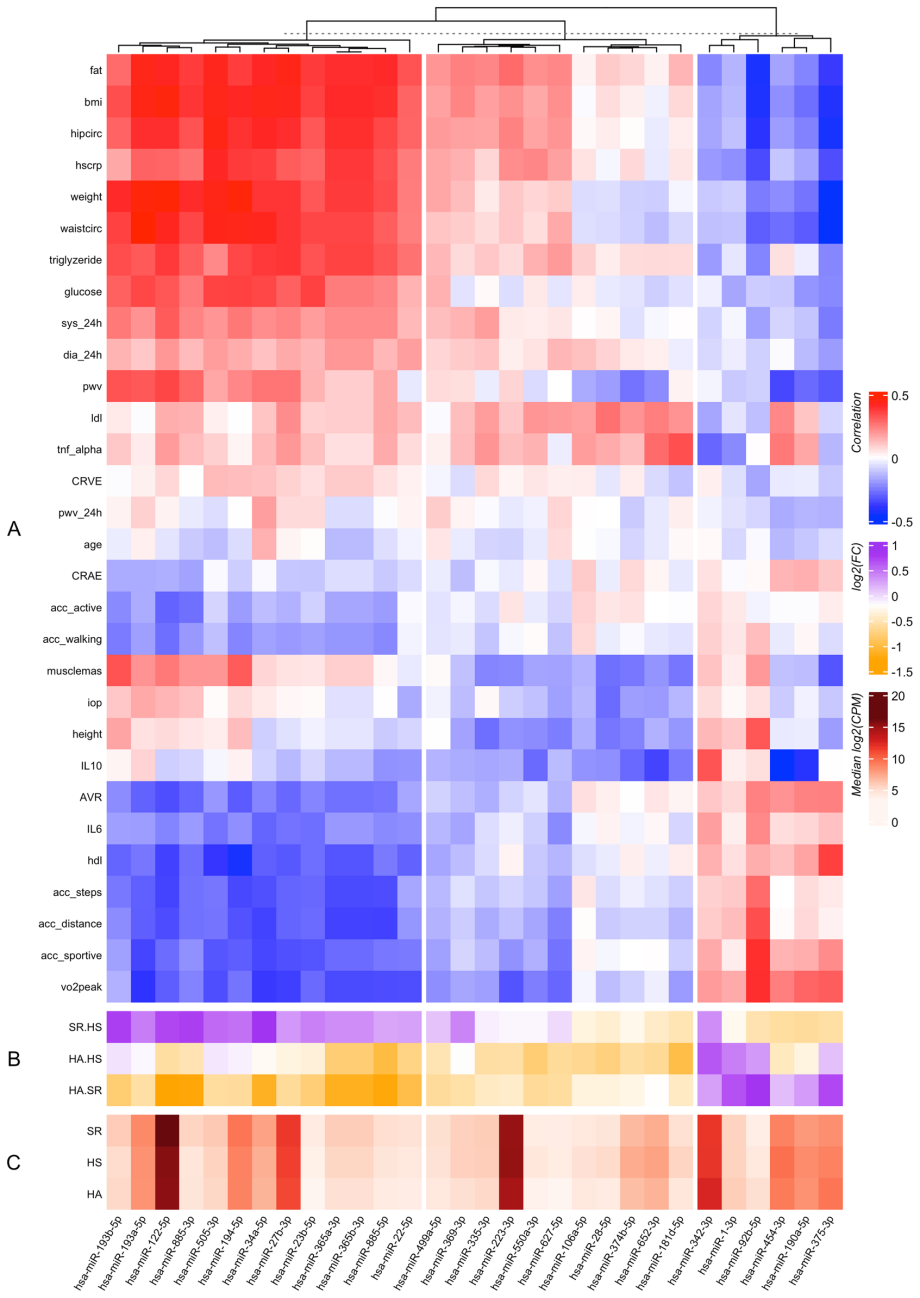
Relationship between miRNA expression levels and values of various physiological parameters. (**A**) Correlation coefficients have been calculated between the miRNA expression levels and the values of the indicated physiological parameters. (**B**) Fold-changes of the corresponding miRNAs among the indicated pairs of samples. (**C**) Median expression level of these miRNAs across samples (in counts per million, CPM). *Fat* fat mass, *BMI* body mass index, *hipcirc* hip circumference, *hscrp* high sensitive C-reactive protein, *waistcirc* waist circumference, *sys_24h* systolic blood pressure based on 24 h monitoring, *dia_24h* diastolic blood pressure based on 24 h monitoring, *pwv* pulse wave velocity, *ldl* low-density lipoprotein, *tnf_alpha* tumor necrosis factor alpha, *CRVE* central retinal venular equivalent, *pwv_24h* pulse wave velocity based on 24 h monitoring, *CRAE* central retinal arteriolar equivalent, *ACC* accelerometer, *active* activity counts, *iop* intraocular pressure, *IL* interleukin, *AVR* arteriolar-to-venular diameter ratio, *hdl* high-density lipoprotein, *VO2peak* peak oxygen uptake, *HA* healthy active individuals, *HS* healthy sedentary individuals, *SR* sedentary individuals with at least 2 cardiovascular risk factors.

To further determine whether specific miRNAs may be linked to biomarkers of CV risk and biological function, we correlated the expression of individual miRNAs with each of these parameters across the entire cohort of participants. Intriguingly, we found distinct clusters of miRNAs to be associated with clusters of CV risk profiles (Fig. 3). miRNAs in the right block of Fig. 3 (miR-342-3p, miR-1-3p, miR-92b-5p, miR-454-3p, miR-190a-5p and miR-375-3p) showed a higher expression in HA compared to SR and, to some extent, also compared to HS (miR-342-3p, miR-1-3p, miR-92b-5p and miR-375-3p). Nearly all of these miRNAs were associated with higher PA levels (acc_steps, acc_distance and/or acc_sportive) and higher VO_2_peak, higher high-density lipoprotein (HDL) and IL6 levels as well as better microvascular health (AVR). In addition, these miRNAs were associated with a lower metabolic risk profile (fat mass, body mass index (BMI), waist- and hip circumference, weight, triglyceride and glucose levels), lower high-sensitive C-reactive protein (hs-crp) levels as well as in some cases, (miR-454-3p, miR-190a-5p and miR-375-3p) with lower pulse wave velocity (PWV).

In contrast, an even larger group of miRNAs in the left block of Fig. 3 showed higher expressions in SR compared to HS and HA (miR-193b-5p, miR-122-5p, miR-885-3p, miR-193a-5p, miR-34a-5p, miR-505-3p, miR-194-5p, miR-27b-3p, miR-885-5p, miR-23b-5b, miR-365a-3p, miR-365b-3p, miR-22-5p). These miRNAs were associated with a higher metabolic risk profile (fat mass, BMI, waist- and hip circumference, weight, triglyceride and glucose levels, 24 h blood pressure) and, in most cases, with higher PWV. In addition, miRNAs in the left block were associated with lower PA (acc_steps, acc_distance, acc_sportive) and CRF levels, lower HDL and IL6 concentrations as well as unfavourable microvascular health (AVR). The miRNAs in the middle block of Fig. 3 showed a pattern of association with cardiovascular parameters that was qualitatively similar but lower in magnitude than the miRNAs in the left block, with increased expression in SR relative to HA.

### Principle component analysis

**Figure 4:**
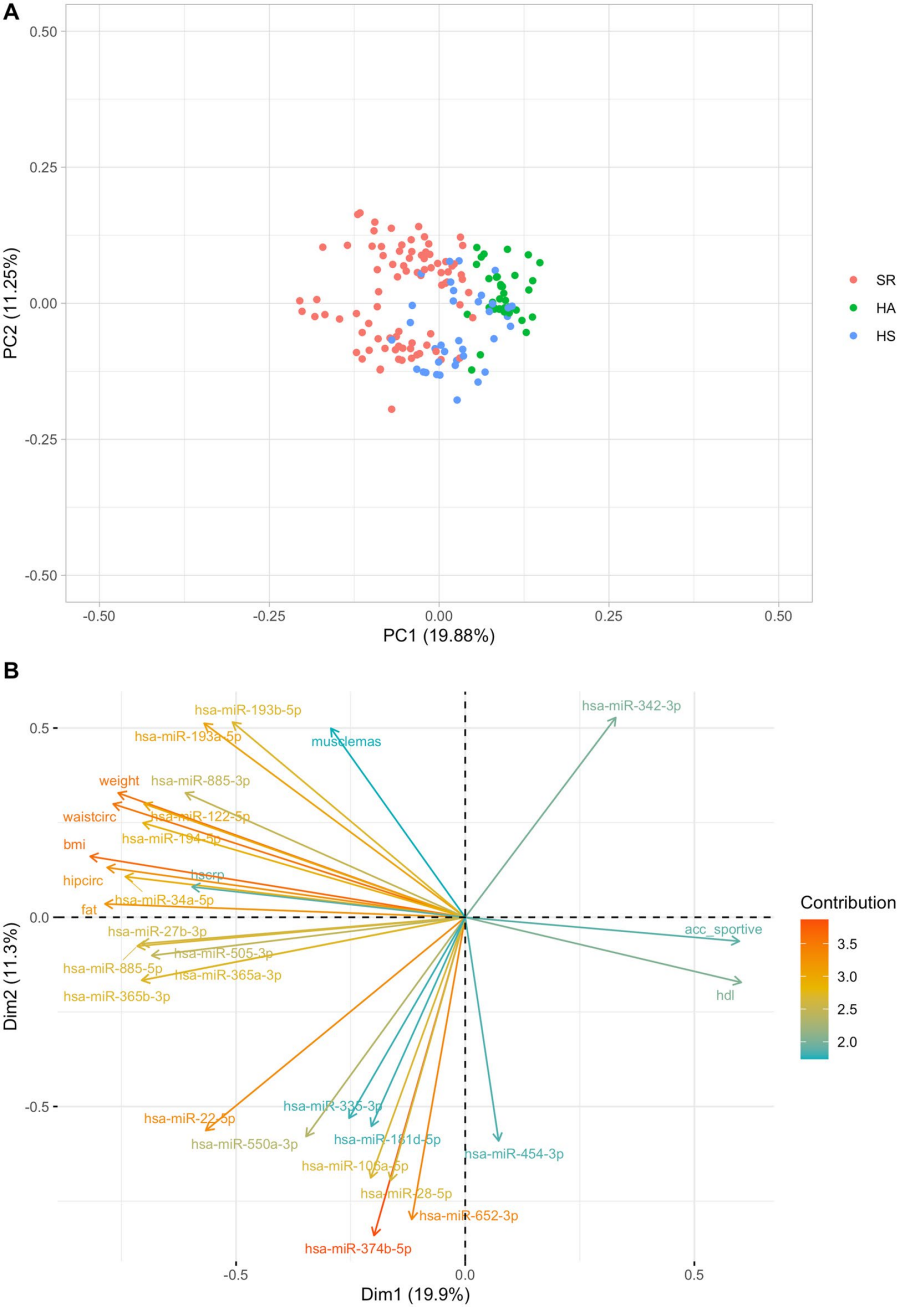
Principal component analysis of the combined miRNA and physiological parameter data. (**A**) Projection of the data on the first two principal components, which explain ~ 20% and 11% of the variance. PC1 imperfectly separates the healthy active individuals from sedentary at risk. (**B**) PCA loadings of the first 30 variables contributing most to explaining the variance in the data (as indicated on the colour scale).

To identify the most informative features defining the three groups, we carried out principal component analyses on the data matrix that included both the differentially expressed miRNAs and the cardiovascular parameters for each participant. The first two principal components explained nearly 20% and 11% of the variance in the data, respectively, the first principal component separating nearly perfectly the HA from the SR samples (Fig. 4A). HS samples were located at intermediate coordinates on PC1. Indeed, by further inspecting the loadings on these PCs, we found that the PC1 axis is defined by the PA status, with sportive walking (acc_sportive) on the positive side of the axis and parameters such as ‘fat’ and ‘bmi’ on the negative side of the axis (Fig. 4B). As expected, many of the miRNAs from the left block in Fig. 3 had similar loadings as the latter set of parameters. No such close relationship was observed between miRNAs and physiological parameters indicating a positive health status. The sole exception to this finding was miR-342-3p. miR-342-5p has previously been identified as an exerkine even if miR-342-3p and miR-342-5p might differ in their functionality^27^. In contrast, PC2 appears to be defined primarily by differences in miRNA expression of disease traits, without specific associations with health status, except of sportive walking and HDL (Fig. 4A,B). By analysing the principal component analyses on the differentially expressed miRNAs without inclusion of the CV risk factors, the first two principal components even explained 27% and nearly 20% of the variance in the data (Supplementary Fig. S2).

## Discussion

Specific circulating miRNAs have previously been described as potential biomarkers of CV risk and disease. Differential expression analysis revealed 30 miRNAs that significantly differed between HA, HS and SR. Increased expression of several of these miRNAs was associated with a higher metabolic risk profile, unfavourable macro- and microvascular health, lower PA and as well as CRF. In contrast, some miRNAs were associated with a lower metabolic risk profile, favourable macro- and microvascular health as well as higher PA and CRF. The first two principal components were found to explain as much as 20% and 11% of the data variance.

### Circulating miRNAs as systemic markers of cardiovascular risk

The majority of miRNAs that exhibited differences in expression among the three individual groups were found to be elevated in the one with elevated CV risk (SR) relative to those of healthy individuals (HA and HS). Higher serum levels of these miRNAs (left block in Fig. 3) were also associated with parameters representing metabolic syndrome, vascular dysfunction as well as lower PA and CRF. Of these, miR-34a-5p, miR-122-5p, miR-27b-3p and miR-885-5p have been identified to play a role in fat and/or lipid metabolism, insulin sensitivity or liver disease. Lowering miR-34a-5p level was previously shown to be an effective strategy to improve stearic-acid-induced lipotoxicity, which plays an important role in beta cell dysfunction and type 2 diabetes^28^. A meta-analysis recently identified miR-34a and miR-122 as biomarkers for non-alcoholic fatty liver and non-alcoholic steatohepatitis^29^. Willeit et al. also highlighted the central role of miR-122, the most abundantly expressed liver miRNA, in the maintenance of lipid and glucose homeostasis, playing a key role in the aetiology of CV and metabolic diseases^30^. Inhibition of miR-27b-3p improved browning capacity of epididymal white adipose tissue, whereas high expressions of miR-27b-3p seemed to inhibit browning ability^31,32^, which led to visceral fat accumulation in mice^31^. Several liver pathologies also seem to be associated with higher levels of miR-885-5p^33^, which supports our findings of higher serum levels in SR compared to both clinically healthy groups and its association to metabolic risk. PCA loadings, described in Fig. 4, indicated that an increase in BMI and related parameters were associated with the release of specific miRNAs into the circulation, notably miR-122 and miR-885, that are known to have unique expression patterns in the liver^34^. In addition to their role in fat and/or lipid metabolism, insulin sensitivity or liver disease, some of the identified miRNAs, in particular miR-34a-5p and miR-122-5p, have also been shown to be upregulated in acute ischemic stroke^35,36^ and acute myocardial infarction^37^. This may indicate a potential role in the regulation of vascular function and the pathogenesis of atherosclerosis. In our study, these miRNAs were associated with markers of micro- and macrovascular function. Higher expression of miR-34a-5p and miR-122-5p were associated with higher PWV and lower AVR, both of these indicating advanced vascular disease and ageing.

Immune response regulation is a key determinant of vascular health. Previous studies showed that miR-885-3p, miR-505-3p and miR-194-5p are principally involved in immune responses. Overexpression of miR-885-3p repressed the pro-inflammatory cytokine production^38^. Up- and/or down-regulation of miR-505-3p and miR-194-5p seem to regulate the transcription of key genes that mediate inflammatory responses^39,40^. Overexpression of these miRNAs was associated with higher inflammation status (hs-CRP), higher CV risk, lower PA and CRF as well as reduced macro- and microvascular health in our study. This may reflect a link between sedentary lifestyle and vascular end organ damage.

Interestingly, miRNAs that are involved in cardiac (miR-23b-5p and miR-365b-3p) or endothelial cell (miR-365b-3p, miR-27b and miR-505-3p) dysfunction were also differentially overexpressed in SR compared to HS and HA. Overexpression of miR-23b-5p has been reported to aggravate cardiac hypertrophy and dysfunction^41^. miR-365b-3p seems to be involved in coronary artery smooth muscle cells proliferation and migration, via its direct target gene ADAMTS1^42^ as induction of endothelial dysfunction^43^. Higher serum levels of miR-27b were observed in patients with peripheral arterial disease^44^ and correlated with disease severity^44^. In addition, miR-27b has previously been shown to be highly expressed in endothelial cells, where it modulates angiogenesis by targeting antiangiogenic genes^45^. Yang et al. showed an overexpression of miR-505 in hypertensive patients. The authors speculated that miR-505 may impair endothelial cell migration^46^. Overexpression of these miRNAs was associated with macro- and microvascular dysfunction in our group of patients with increased CV risk, which indicated their sensitivity to detect individual vascular end organ damage.

### Circulating miRNAs as systemic markers of cardiovascular health

The identification of specific miRNAs associated with PA and CRF can provide new epigenetic insights into exercise-related vasoprotection and risk reduction^20^. The right block in Fig. 3 is composed of four miRNAs: miR-342-3p, miR-1-3p, miR-92b-5p and miR-375-3p that were more abundant in HA compared to both sedentary groups, with (SR) or without (HS) CV risk. These miRNAs were also associated with higher PA and CRF levels as well as favourable vascular health. Higher PA and CRF have previously been highlighted as important components of prevention strategies for non-communicable diseases^47^. Several studies identified circulating miRNAs that seem to be involved in acute or chronic exercise responses and highlighted their potential role in disease prevention^17,48^. Our study thus helps identify miRNAs that are potential circulating biomarkers reflecting the protective effect of exercise on vascular health. Ray et al. showed that miR-342-3p has anti-inflammatory properties and showed its potential as a subclinical CV disease biomarker^49^. Hou and colleagues highlighted miR-342-5p as novel exerkine mediating cardioprotection^27^. In contrast, overexpression of miR-1-3p and miR-92b-5p have previously been associated with cardiac damage. For example, miR-1-3p seems to be overexpressed in acute myocardial infarction^50,51^. Higher miR-92b-5p expression was found in decompensated dilated cardiomyopathy^52^ and HFrEF^53^ patients showing positive correlations with left atrial diameter, left ventricular systolic and diastolic diameter, as well as a negative correlations with left ventricular fraction shortening and left ventricular ejection fraction^52,53^. In our study, HA were, by definition, free of any cardiac diseases and were characterized by high PA and CRF levels. Therefore, our data indicate that miR-1-3p and miR-92b-5p may also reflect cardiac adaptations in response to an active lifestyle, and not necessarily cardiac damage per se. miR-375-3p is also involved in cardiac remodelling as myotrophin, a direct target gene of miR-375-3p^54^, has been shown to initiate the transition from cardiac hypertrophy to heart failure^55–57^. Further research is needed to investigate the underlying mechanisms of these three miRNAs with respect to the cardiac phenotype in health and disease. It still needs to be verified whether the above-mentioned miRNAs may indeed mediate the protective effect of higher PA and CRF on vascular and cardiac health.

### Circulating miRNAs and their target genes

The miRNAs differentiated in our study have hundreds of target genes that are involved in several physiological as well as pathophysiological processes. The regulation of target organ function is complex and the underlying mechanisms are multifactorial. Due to the fact that the targets of the highlighted miRNAs exert their function in various human tissues that we could not sample in the scope of this study, we are not able to bridge the gap between predicted regulation and phenotype with the available data. Nevertheless, to prioritize future analyses we summarized all genes predicted to be targeted by at least three miRNAs described in Fig. 3 (Supplementary Table S2). A network of target genes is more likely to be a key regulator of target organ function than single target gene pathways. Protein–protein association networks can support functional discovery in genome-wide experimental datasets^58^. As a hypothesis for relevant underlying networks, we therefore analysed two STRING networks^59^ including the top ten target genes of miRNAs highlighted in Fig. 3. These two networks are described in Supplementary Figs. S3 and S4. Supplementary Figure S3 describes target gene interactions associated with CV risk. Interestingly, miRNAs associated with CV risk are associated with target genes that are known as regulators of several cellular processes such as cell proliferation, differentiation or migration (CTNNB1, FBXW7, PDGFRA and SOS1) or regulators of cardiac remodelling (FOXP1)^60,61^. The top ten target genes of miRNAs associated with healthy lifestyle showed lower degrees of interactions with a smaller network of target genes. As an example, target gene HDAC7 has been shown to regulate myocyte enhancer factor-2, which is a mediator of skeletal muscle differentiation^62^. Activation of this target gene may be a potential response mechanism to regular exercise in healthy active individuals (HA) compared to both sedentary groups (HS and SR). The above target gene networks are based on GeneCards® (Weizmann Institute of Science, v5.3.0 Build 405) but remain hypothetical in nature and are not discussed in further detail. They are presented only as examples for the complexity of miRNA-induced target gene networks and their potential regulatory impact on end organ function.

### Limitations

This cross-sectional study is only associative in nature. Long-term and larger-scale follow-up studies are needed to investigate the predictive value of specific targeted miRNAs as sensitive biomarkers to detect metabolic or vascular disease risk, accounting for main confounding factors. Due to the relatively small sample size per group, we refrained from applying adjustments for covariates in our statistical approach, which has to be considered when interpreting the results. Our study was not designed to offer detailed mechanistic insights and has clear limitations with respect to causal correlations. This will have to be elucidated in future studies with the primary aim to clarify causal associations and the mechanisms involved. In addition, intervention studies are of interest to elaborate on potential treatment targets. Future studies need to further clarify the underlying mechanisms and systemic impact of the miRNAs we identified in an untargeted approach. This includes the validation of the differential expression of target genes across the transcriptional and translational landscape, which was beyond the scope of our study. Exploration of pathways from miRNA binding to mRNA transcription to protein translation and end organ function, which may prove to be an ambitious undertaking due to the complexity of the networks involved, need to be prioritized in future research to better understand the underlying epigenetic mechanisms of cardiovascular disease.

## Conclusion

Our study employed an untargeted approach, profiling miRNA expression in serum by next generation sequencing. We have identified 30 miRNAs associated with cardiovascular health and physical activity or disease in an older population. Most of these miRNAs were associated with higher metabolic risk, reduced macro- and microvascular health and low PA and CRF as biomarkers of CV risk and disease. Fewer miRNAs were associated with low metabolic risk, beneficial macro- and microvascular health as well as high PA and CRF as markers of good CV health. It can be postulated that a complex network of target genes is involved in the regulation of cardiovascular processes and organ function. Further research is warranted to investigate whether the miRNAs identified by our untargeted approach are putative targets that have the potential to translate into novel prevention and treatment strategies of CV disease. Moreover, the identification of relevant miRNAs in our study needs further validation and support by further downstream analysis in future studies.

## Supplementary Information

Supplementary Information.

## Data Availability

The datasets generated during the current study are available from the corresponding author on reasonable request.
