# Supplementary material for: Untargeted sequencing of circulating microRNAs in a healthy and diseased older population
Source: Sci Rep. 2022 Feb 22;12:2991. doi: 10.1038/s41598-022-06956-4 (PMC8863825; doi:10.1038/s41598-022-06956-4)
Supplement: Supplementary file 1 — Supplementary Information. [file 41598_2022_6956_MOESM1_ESM.docx]

**SUPPLEMENTARY MATERIAL**

**Untargeted sequencing of circulating microRNAs in a healthy and diseased older population**

Lukas Streese^1^, Philippe Demougin^2^, Paula Iborra^3^, Alexander Kanitz^3^, Arne Deiseroth^1^, Julia M. Kröpfl^1^, Arno Schmidt-Trucksäss^1^, Mihaela Zavolan^3^, Henner Hanssen^1^*

^1^Department of Sport, Exercise and Health, Medical Faculty, University of Basel, Basel, Switzerland.

^2^Transfaculty Research Platform Molecular and Cognitive Neurosciences, Life Sciences Training Facility, Biozentrum, University of Basel, Basel, Switzerland.

^3^Computational and Systems Biology, Biozentrum, University of Basel, Basel, Switzerland.

**Short title**: Untargeted sequencing of circulating miRNAs

***Address for correspondence:**Prof. Henner Hanssen, FESC
Professor of Preventive Sports Medicine
Department of Sport, Exercise and Health, University of Basel
Birsstrasse 320 B , 4052 Basel
Tel. 041 (0)61 377 8746|Fax 041 (0)61 207 4748
email: henner.hanssen@unibas.ch


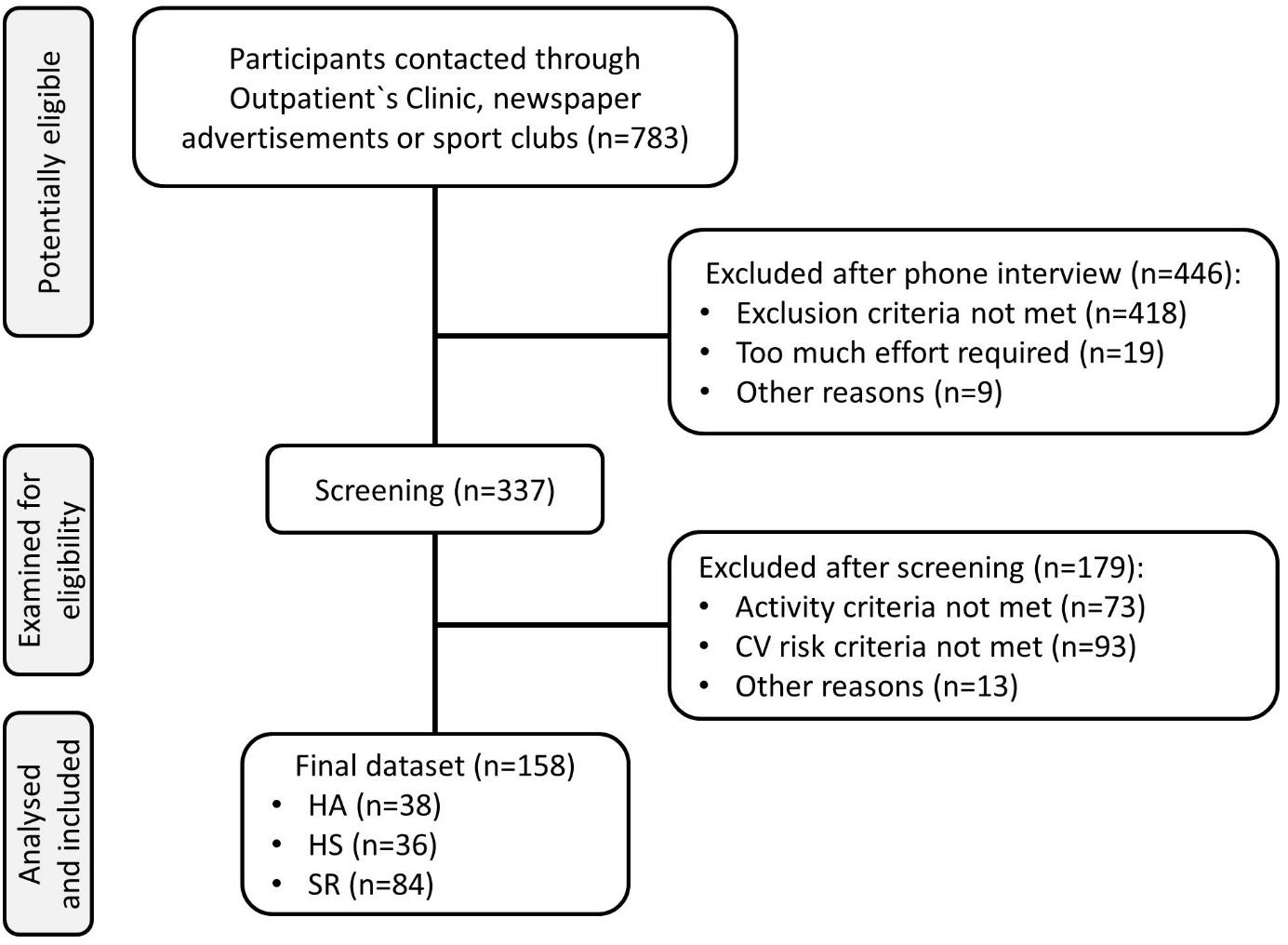


**Figure S1. Flow-chart**

CV, cardiovascular; HA, healthy active, HS, healthy sedentary, SR, sedentary patients with increased cardiovascular risk.


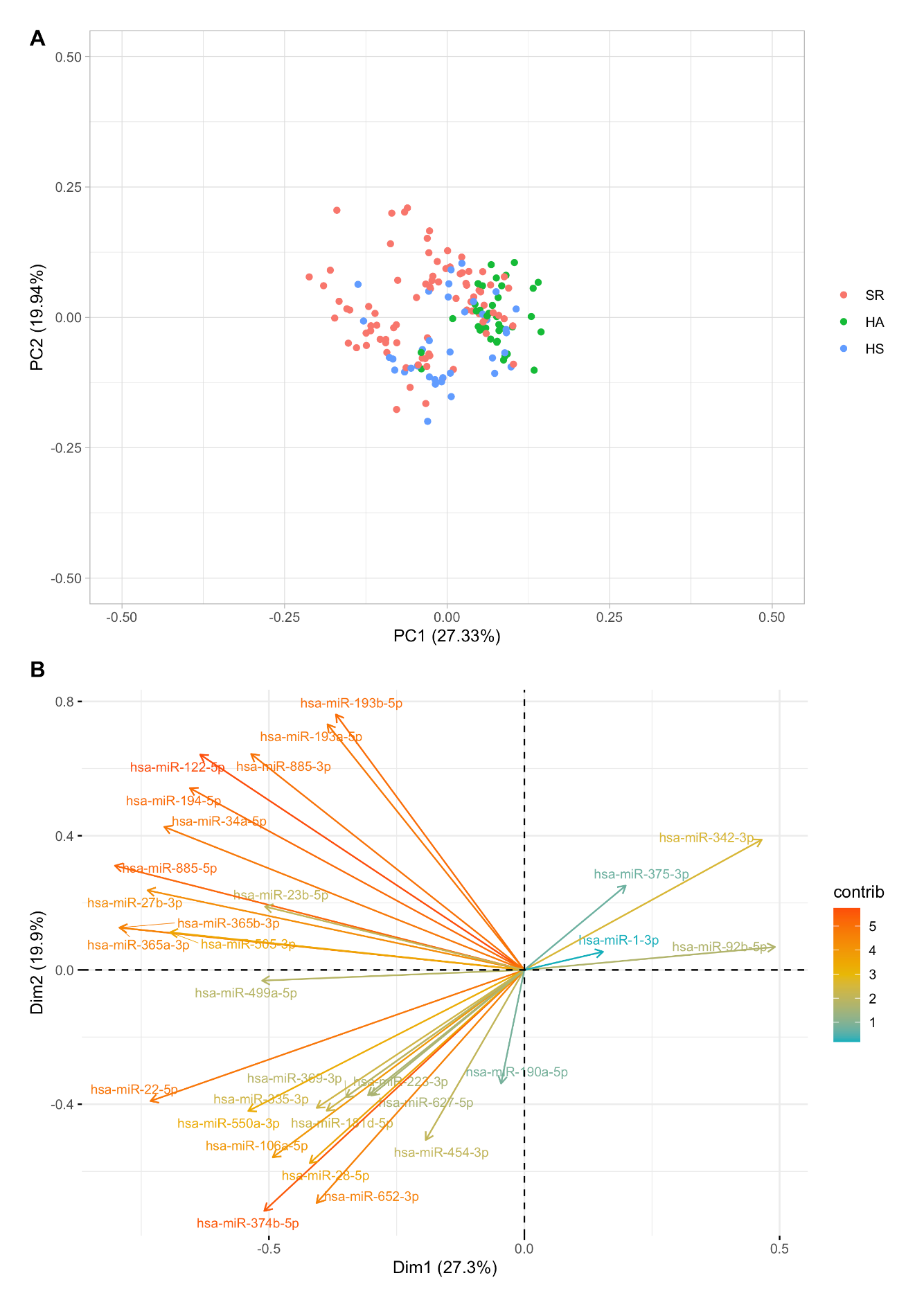


**Figure S2. Principal component analysis of the differentially expressed miRNAs only.**

(A) Projection of the data on the ﬁrst two principal components, which explain 27% and ~20% of the variance. (B) PCA loadings of all 30 deferentially expressed miRNAs with its contribution to the explained variance in the data (as indicated on the colour scale).


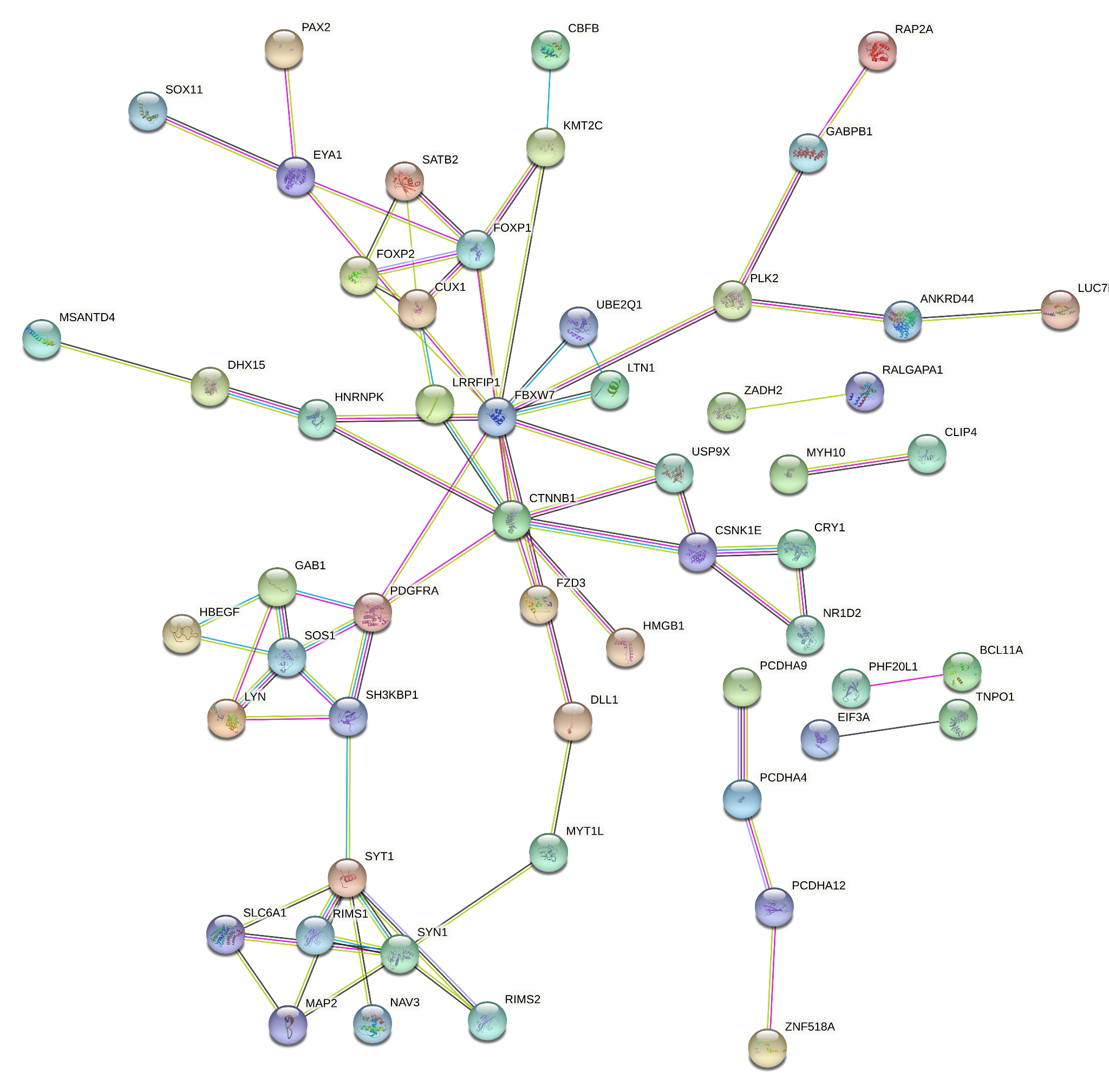


**Figure S3. STRING network of top ten target genes associated with cardiovascular disease**

Top ten target genes of miRNAs highlighted in Figure 3 (left block) were included in this STRING network. Color saturation of the edges represents the confidence score of the functional association. Red lines, indicates the presence of fusion evidence; green lines, neighborhood evidence; blue lines, cooccurrence evidence; purple lines, experimental evidence; yellow lines, text-mining evidence; light blue lines, database evidence; black lines, co-expression evidence.


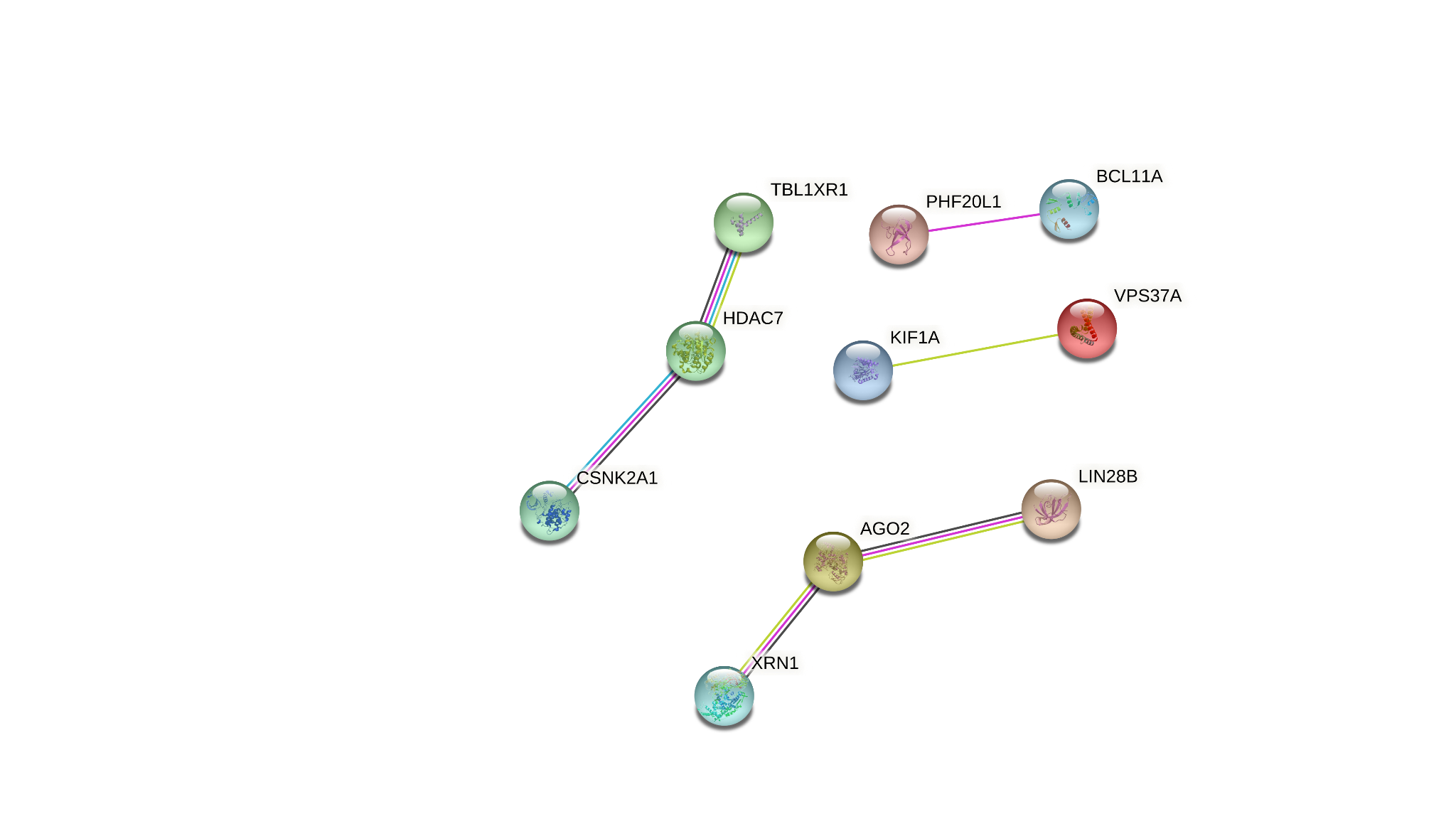


**Figure S4. STRING network of top ten target genes associated with a healthy lifestyle**

Top ten target genes of miRNAs highlighted in Figure 3 (right block) were included in this STRING network. Color saturation of the edges represents the confidence score of a functional association. Red lines, indicates the presence of fusion evidence; blue lines, cooccurrence evidence; purple lines, experimental evidence; yellow lines, text-mining evidence; light blue lines, database evidence.

**Table S1. Risk factors.**

| **Risk factor** | **Definition** | **SR**  **n (%)** |
| --- | --- | --- |
| **Obesity** | body mass index ≥30 kg/m^2^ | 71 (85) |
| **Hypertension** | ≥140 mmHg systolic or ≥90 mmHg diastolic BP during 24h monitoring or  treatment with antihypertensive medications | 18 (21)  38 (45) |
| **Diabetes** | fasting glucose ≥5.6 mmol/l or  antidiabetic medications | 24 (29)  10 (12) |
| **Smoking** | current smoking status | 28 (33) |
| **Low HDL** | <1.0 mmol/l (male); <1.2 mmol/l (female) | 28 (33) |
| **Hypertriglyceridemia** | triglyceride >1.7 mmol/l | 24 (29) |
| **High LDL** | >4.9 mmol/l or  cholesterol lowering drugs | 2 (2)  15 (18) |

SR, sedentary patients with increased cardiovascular risk; BP, blood pressure; HDL, high-density lipoprotein;
LDL, low-density lipoprotein.

**Table S2. Summary of target genes triggered by ≥ three miRNAs described in Figure 3.**

| **Target gene** | **Gene name** | **miRNAs** |
| --- | --- | --- |
| FAM126B | family with sequence similarity 126 member B | miR-342-3p, miR-885-3p, miR-34a-5p, miR-505-3p, miR-27b-3p |
| MSL2 | male-specific lethal 2 homolog (Drosophila) | miR-34a-5p, miR-27b-3p, miR-885-5p, miR-23b-5 |
| CREBRF | CREB3 regulatory factor | miR-34a-5p, miR-505-3p, miR-27b-3p |
| KITLG | KIT ligand | miR-34a-5p, miR-505-3p, miR-27b-3p, miR-194-5 |
| MSANTD3 | Myb/SANT-like DNA-binding domain containing 3 | miR-92b-5p, miR-342-3p, miR-23b-5p |
| LIN28B | lin-28 homolog B | miR-885-3p, miR-27b-3p, miR-194-5 |
| CADM1 | cell adhesion molecule 1 | miR-505-3p, miR-27b-3p, miR-194-5 |
| SORCS1 | sortilin-related VPS10 domain containing receptor 1 | miR-193b-5p, miR-122-5p, miR-885-5p |
| CHD1 | chromodomain helicase DNA binding protein 1 | miR-885-3p, miR-34a-5p, miR-194-5 |
| ZBTB34 | zinc finger and BTB domain containing 34 | miR-193b-5p, miR-27b-3p, miR-885-5p |
| FLRT3 | fibronectin leucine rich transmembrane protein 3 | miR-885-3p, miR-505-3p, miR-27b-3p |
| MEIS2 | meis homeobox 2 | miR-505-3p, miR-27b-3p, miR-194-5 |
| SOX6 | SRY-box transcription factor 6 | miR-342-3p, miR-505-3p, miR-194-5 |
| GALNT7 | polypeptide N-acetylgalactosaminyltransferase 7 | miR-885-3p, miR-34a-5p, miR-27b-3p |
| MTMR4 | myotubularin related protein 4 | miR-27b-3p, miR-885-5p, miR-23b-5 |
| FOXP1 | forkhead box P1 | miR-193b-5p, miR-122-5p, miR-34a-5p, miR-885-5p |
| FOXP2 | forkhead box P2 | miR-193b-5p, miR-122-5p, miR-34a-5p, miR-27b-3p |
| FBXO33 | F-box protein 33 | miR-193b-5p, miR-27b-3p, miR-885-5p |
| TMEM185B | transmembrane protein 185B | miR-193b-5p, miR-885-5p, miR-23b-5 |
| LCOR | ligand dependent nuclear receptor corepressor | miR-193b-5p, miR-27b-3p, miR-23b-5 |
| DIXDC1 | DIX domain containing 1 | miR-34a-5p, miR-885-5p, miR-23b-5 |
| PDE7A | phosphodiesterase 7A | miR-193b-5p, miR-122-5p, miR-34a-5p, miR-23b-5 |
| CDK14 | cyclin-dependent kinase 14 | miR-193b-5p, miR-27b-3p, miR-885-5p |
| PLAG1 | pleiomorphic adenoma gene 1 | miR-122-5p, miR-34a-5p, miR-27b-3p |
| SATB1 | SATB homeobox 1 | miR-193b-5p, miR-122-5p, miR-34a-5p |
| NPAS3 | neuronal PAS domain protein 3 | miR-122-5p, miR-27b-3p, miR-885-5p |
